# MiR-873-5p regulated LPS-induced oxidative stress *via* targeting heme oxygenase-1 (HO-1) in KGN cells

**DOI:** 10.1039/c8ra06697c

**Published:** 2018-11-22

**Authors:** Hui Zhang, Zhengnan Gao, Yanjie Zhang, Huihui Wang, Yongfeng Li

**Affiliations:** Department of Endocrinology, The First Affiliated Hospital of Henan Polytechnic University, The Second People's Hospital of Jiaozuo No. 17 Minzhu South Road Jiaozuo 454000 China yfli0322@126.com +86 03912625785 +86 03912631994; Department of Endocrinology, Dalian Municipal Centre Hospital Dalian 116033 China

## Abstract

Polycystic ovary syndrome (PCOS) is one of the most common endocrine disorders in women. Increasing evidence reveals that PCOS may be associated with an increased level of oxidative stress. This study aimed to explore the role and potential mechanism of microRNA-873-5p (miR-873-5p) in PCOS. Quantitative real time-PCR (qRT-PCR) analysis was performed to evaluate the miR-873-5p levels in both clinical follicular fluid samples from patients with PCOS and cultured human ovarian granulosa cell-like KGN cells. The results indicated that miR-873-5p was up-regulated in the follicular fluid from patients with PCOS, as well as in the LPS-induced KGN cells. MTT assay showed that miR-873-5p inhibitor attenuated the LPS-induced inhibition of KGN cell viability. Flow cytometry indicated that miR-873-5p inhibitor suppressed cell apoptosis in LPS-induced KGN cells. Besides, miR-873-5p inhibitor resulted in a decrease in oxidative stress, which was evidenced by the reduced production of reactive oxygen species (ROS) and malondialdehyde (MDA). Further luciferase reporter assay proved that miR-873-5p directly targeted the 3′UTR of HO-1 mRNA. Small-interfering RNA (siRNA) targeting heme oxygenase-1 (HO-1) attenuated the effect of miR-873-5p inhibitor on oxidative stress in KGN cells. Besides, we also found that miR-873-5p inhibitor activated the p38/Nrf2/HO-1 signaling pathway in KGN cells. The findings may provide insightful evidence for preventing and treating PCOS by targeting miR-873-5p.

## Abstract 2

Polycystic ovary syndrome (PCOS) is one of the most common endocrine disorders in women.

## Graphical abstract

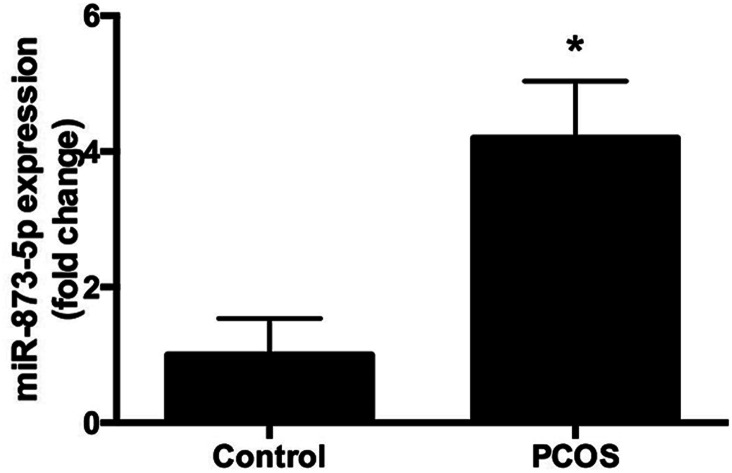

## 1. Introduction

Polycystic ovary syndrome (PCOS) is one of the most common endocrine disorders and affects 4–12% of women worldwide, leading to poor fertility.^1^ The risk factors of PCOS include genetic factors, obesity, and lack of exercise.^2^ There is no effective therapy approach for PCOS, and the most suggested method is changing lifestyle such as weight loss and exercise.^3^ Recently, increasing evidence has revealed that PCOS may be associated with an increased level of oxidative stress and chronic inflammation.^4^ Furthermore, it has been reported that a high level of oxidative stress in PCOS is considered as an incentive for the risk of cancer.^4^ Therefore, targeting oxidative stress and inflammation in PCOS might be a new approach for the prevention and treatment of PCOS.

MicroRNA (miRNA) is a group of small non-coding RNA molecules that contain about 22 nucleotides.^5^ MiRNAs exert their cellular functions *via* targeting the 3′UTR of the mRNAs, thus regulating the expressions of target genes.^5^ MiRNAs have been implicated with many human diseases such as cancers and inflammatory related diseases, and therefore pursued as clinical diagnostic markers and therapeutic targets for the diseases.^5,6^ In recent years, aberrant expressions of miRNAs have been observed in PCOS patients. Those miRNAs might serve as potential biomarkers for the diagnosis and a new approach for PCOS treatment.^7,8^ MiR-873 has been reported to be expressed in dominant follicles and is involved in follicular cell proliferation and steroidogenesis.^9,10^ Besides, miR-873 is predominantly expressed in mural granulosa cells, and it may play a potential role in regulation of granulosa cells during follicles maturation.^9^ Additionally, Liu *et al.* reported that miR-873 could be induced by interleukin 17 (IL-17) in experimental autoimmune encephalomyelitis (EAE) mice.^11^ The induced miR-873 promotes the production of inflammatory cytokines, indicating miR-873 participates in the inflammatory damage in EAE mice.^11^ However, the role of miR-873 in PCOS has not been reported yet.

LPS, the major component of the outer membrane of Gram-negative bacteria, is widely used to induce oxidative stress and inflammation model in the *in vivo* and *in vitro* experiments.^12,13^ Previous study reported that *in vitro* model of PCOS could be established using LPS stimulation in KGN cells.^14^ Zuo *et al.* reported that iridoids with genipin stem nucleus inhibit LPS-induced inflammation and oxidative stress in KGN cells, suggesting that iridoids may have potential roles in attenuating PCOS complications.^14^ In the present study, we examined the miR-873 levels in both clinical samples from PCOS patients and cultured human ovarian granulosa cell-like KGN cells. We further investigated the role of miR-873-5p in LPS-induced KGN cells and explored the potential mechanism.

## 2. Materials and methods

### 2.1 Clinical samples

The clinical samples were collected from 20 patients with PCOS and 20 patients without PCOS who underwent *in vitro* fertilization/embryo transfer (IVF/ET) due to infertility at the First Affiliated Hospital of Henan Polytechnic University. Patients were excluded if they have diabetes, uncontrolled hypertension, hypothyroidism, hyperthyroidism, current cigarette smokers, liver/renal disease, or cardiovascular disease. Follicular fluid was carefully collected from follicles >15 mm in diameter. Follicular fluid was excluded when contaminated with blood. Follicular fluid were centrifuged at 5000*g* at 4 °C for 10 min and the cellular pellets were collected for further analysis. All participants have written informed consent. The present study was approved by the ethics committee of the First Affiliated Hospital of Henan Polytechnic University (Approval number: No. 284 534).

### 2.2 Cell culture and treatment

The KGN cells were obtained from Suer Biotech (Shanghai, China). The cells were maintained in Dulbecco's Modified Eagle Medium (DMEM)/F-12 medium (Gibco, Carlsbad, CA) containing 10% FBS (Gibco), penicillin G (100 U ml^−1^, Invitrogen, Carlsbad, CA, USA), and streptomycin sulfate (0.1 mg ml^−1^, Invitrogen). Then the cells were incubated in a humidified atmosphere with 5% CO_2_ at 37 °C. For some experiments, the cells were incubated with 2 μg ml^−1^ LPS and/or SB203580 for 24 h.

### 2.3 Cell transfection

Specific inhibitor of miR-873-5p, inhibitor control, the small interfering RNA (siRNA) targeting HO-1 (HO-1 siRNA), control siRNA (GenePharma Co. Ltd, Shanghai, China), HO-1 overexpressing plasmid (pcDNA–HO-1) or empty vector (pcDNA) were transfected into KGN cells using Lipofectamine 2000 (Invitrogen) according to manufacturer's protocol. The HO-1 overexpressing plasmid was constructed by inserting the coding sequence of HO-1 into pcDNA3.1 vector (Thermo Scientific). After 48 h transfection, the cells were collected for further experiments.

### 2.4 Quantitative real time-PCR (qRT-PCR)

Total RNA was prepared using Trizol reagent (Invitrogen). Then total RNA (1 μg) was reverse transcribed using the PrimeScript™ RT Reagent Kit (Takara Bio, Shiga, Japan) following the manufacturer's instructions. The qRT-PCR was performed using SYBR® Premix Ex Taq™ (Takara) or MicroRNAs Quantitation PCR Kit (Sangon Biotech, Shanghai, China). PCR was run on Realtime PCR system (Applied Biosystems, Carlsbad, CA) under the following condition: initial denaturation at 95 °C for 5 min, 95 °C for 20 s, annealing at 56 °C for 30 s, extension at 72 °C for 1 min, 35 cycles, and final extension at 72 °C for 5 min. Fold changes in expression of each gene were calculated using the 2^−ΔΔ*C*_t_^ method. And the *C*_t_ values for the mRNA and miR-873-5p were normalized to β-actin and U6, respectively.

### 2.5 Cell viability detection

Cell viability of KGN cells was determined by the 3-(4,5-dimethylthiazol-2-yl)-2,5-diphenyltetrazolium bromide (MTT) assay. KGN cells with different transfections were seeded in 96-well plates at a density of 5 × 10^3^ cells per well. The cells were incubated at 37 °C for 24 h in the presence or absence of LPS (2 μg ml^−1^) and/or SB203580. After incubation, 10 μl of MTT solution (5 mg ml^−1^) was added to each well and then incubated for another 4 h at 37 °C. Subsequently, 150 μl of dimethyl sulfoxide (DMSO) was added to each well and slightly shook for 10 min. Finally, the optical density (OD) at 570 nm was measured using an spectrophotometer (Bio-Rad, Philadelphia, PA, USA).

### 2.6 Measurement of intracellular reactive oxygen species (ROS) and malondialdehyde (MDA) production

The ROS levels of KGN cells were quantified using the DCFH-DA with a ROS Assay Kit (Beyotime Biotech, Shanghai, China) according to the manufacturer's protocols. The MDA production was detected using Lipid Peroxidation MDA Assay Kit obtained from Beyotime Biotech as described by the manufacture's instruction. After different treatments, the KGN cells were lysed using RIPA lysis buffer (Beyotime Biotech), and the supernatant was collected. Thiobarbituric acid (TBA) solution (200 μl) was added to 100 μl supernatant, and the mixture was heated for 15 min at 100 °C. After centrifugation at 1000*g* for 10 min, the absorbance at 532 nm was determined.

### 2.7 Western blot

Total proteins of KGN cells were extracted using RIPA lysis buffer (Beyotime Biotech). The nuclear proteins were extracted using a Nucleoprotein Extraction Kit (Sangon Biotech). Protein concentrations were determined using a BCA protein assay kit (Boster, Wuhan, China). Then the proteins were separated by 10% SDS-PAGE and blotted onto polyvinylidene difluoride (PVDF) membranes (Millipore, Billerica, MA, USA). After blocking with 5% non-fat milk solution for 1 h, the membranes were incubated with primary antibodies against HO-1 (1: 500, Abcam, Cambridge, MA, USA), p-p38 (1: 1000, Cell Signaling Technology, MA, USA), p38 (1: 1000, Cell Signaling Technology), Nrf2 (1: 500, Abcam), lamin B (1: 500, Abcam) and β-actin (1: 500, Abcam) at 4 °C overnight. Then the membranes were incubated with HRP-conjugated secondary antibodies (1: 5000, Cell Signaling Technology) for 1 h at room temperature. Finally, the signals were detected using an enhanced chemiluminescent (ECL) reagent (Thermo Scientific, Waltham, MA, USA). The intensity of bands was calculated using software Image J (NIH, USA).

### 2.8 Cell apoptosis detection

Cell apoptosis was analyzed by flow cytometry using an annexin V/propidium iodide (PI) staining kit (Sigma). Briefly, KGN cells with different treatments were stained with Annexin V-FITC and propidium iodide (PI) for 15 min at room temperature in the dark. Then the cells were subjected to flow cytometry analysis using a FACSCalibur (Becton Dickinson, San Jose, CA, USA).

### 2.9 Luciferase reporter assay

The 3′UTR fragment of HO-1 (HO-1-WT) containing the conserved binding sites for miR-873-5p was amplified by PCR. The mutant 3′UTR fragment of HO-1 (HO-1-MUT) was obtained by the point mutation method using the KOD-Plus mutagenesis kit (Toyobo, Ohtsu, Japan). The HO-1-WT and HO-1-MUT fragments were inserted into Bgl II/BamHI digested pGL3 promoter luciferase vector (Promega, Madison, WI). Then the HO-1-WT or HO-1-MUT vector was co-transfected with miR-873-5p mimics or control mimics into 293T cells using lipofectamine 2000 (Invitrogen). After 48 h, the luciferase activity was detected using a dual-luciferase reporter assay system (Promega).

### 2.10 Statistical analysis

The results are presented as the means ± SD of three independent experiments. Statistical significance was assessed using the GraphPad Prism 5 software (GraphPad Software, San Diego, CA). Comparisons between two groups were analyzed using student's *t*-test; comparisons among more than two groups were performed using one-way ANOVA. A *p* value less than 0.05 was considered to be statistically significant.

## 3. Results

### 3.1 MiR-873-5p was up-regulated in the PCOS samples and LPS-induced KGN cells

**Fig. 1:**
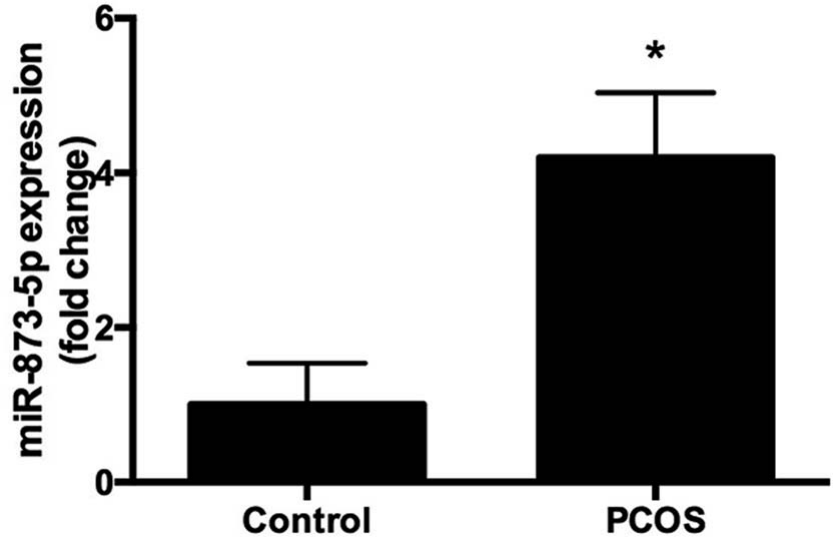
MiR-873-5p was up-regulated in the patients with PCOS. The follicular fluid samples from 20 patients with PCOS and 20 patients without PCOS were collected. The miR-873-5p levels were measured using qRT-PCR. *p < 0.05 *vs.* control group (patients without PCOS).

**Fig. 2:**
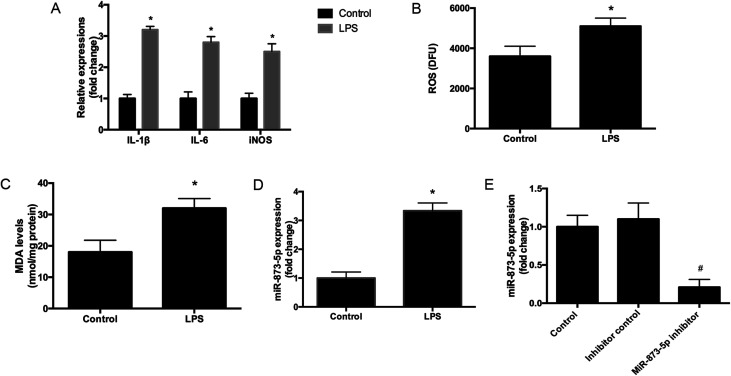
MiR-873-5p was up-regulated in the LPS-induced KGN cells. (A) The mRNA levels of IL-1β, IL-6, and iNOS were measured by RT-PCR. (B) ROS levels in KGN cells. (C) MDA levels in KGN cells. (D) The expression of miR-873-5p in KGN cells. (E) The expression of miR-873-5p in KGN cells at 48 h after transfection with miR-873-5p inhibitor or inhibitor control. *p < 0.05 *vs.* control. ^#^*p* < 0.05 *vs.* inhibitor control.

Compared to the patients without PCOS, the miR-873-5p levels were significantly up-regulated in follicular fluid from patients with PCOS (Fig. 1). Next, we aimed to evaluate the role of miR-873-5p in an *in vitro* PCOS model. We used LPS to stimulate inflammation response and oxidative stress in KGN cells. After stimulation, the mRNA levels of IL-1β, IL-6, and iNOS were measured by RT-PCR. As shown in Fig. 2A, LPS induction led to a significant increase in mRNA expressions of IL-1β, IL-6, and iNOS in KGN cells. LPS also induced the production of ROS and MDA in KGN cells (Fig. 2B and C). The expression of miR-873-5p in LPS-induced KGN cells was dramatically increased when compared with the control cells (Fig. 2D). To investigate the role of miR-873-5p in LPS-induced KGN cells, the miR-873-5p inhibitor was transfected into KGN cells. As shown in Fig. 2E, the miR-873-5p expression was markedly inhibited by the miR-873-5p inhibitor transfection.

### 3.2 MiR-873-5p inhibitor attenuated the LPS induced oxidative stress and cell apoptosis in KGN cells

**Fig. 3:**
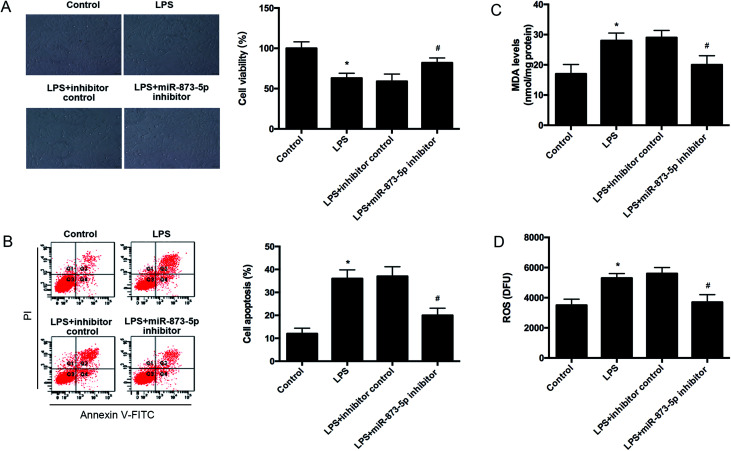
MiR-873-5p inhibitor attenuated the LPS induced oxidative stress and cell apoptosis in KGN cells. KGN cells was transfected with miR-873-5p inhibitor or inhibitor control, and then incubated in the presence or absence of LPS for 24 h. (A) Cell viability was measured by MTT assay. (B) Cell apoptosis was detected by flow cytometry. (C) MDA levels in KGN cells. (D) ROS levels in KGN cells. *p < 0.05 *vs.* control; ^#^*p* < 0.05 *vs.* LPS + inhibitor control.

Then we detected the cell viability of KGN cells using MTT assay. We found that LPS caused an obvious decrease in cell viability, while the reduction was attenuated by miR-873-5p inhibitor (Fig. 3A). Besides, the effect of miR-873-5p inhibitor on cell apoptosis was also measured. The flow cytometry indicated that LPS induced the cell apoptosis, whereas, the induction was alleviated by miR-873-5p inhibitor (Fig. 3B). Moreover, the ROS and MDA production were measured to reflect the oxidative stress level. Compared with the control group, the ROS and MDA production in LPS-induced KGN cells were significantly increased. The ROS and MDA production induced by LPS were reduced by miR-873-5p inhibitor (Fig. 3C and D).

### 3.3 MiR-873-5p directly targeted to HO-1

**Fig. 4:**
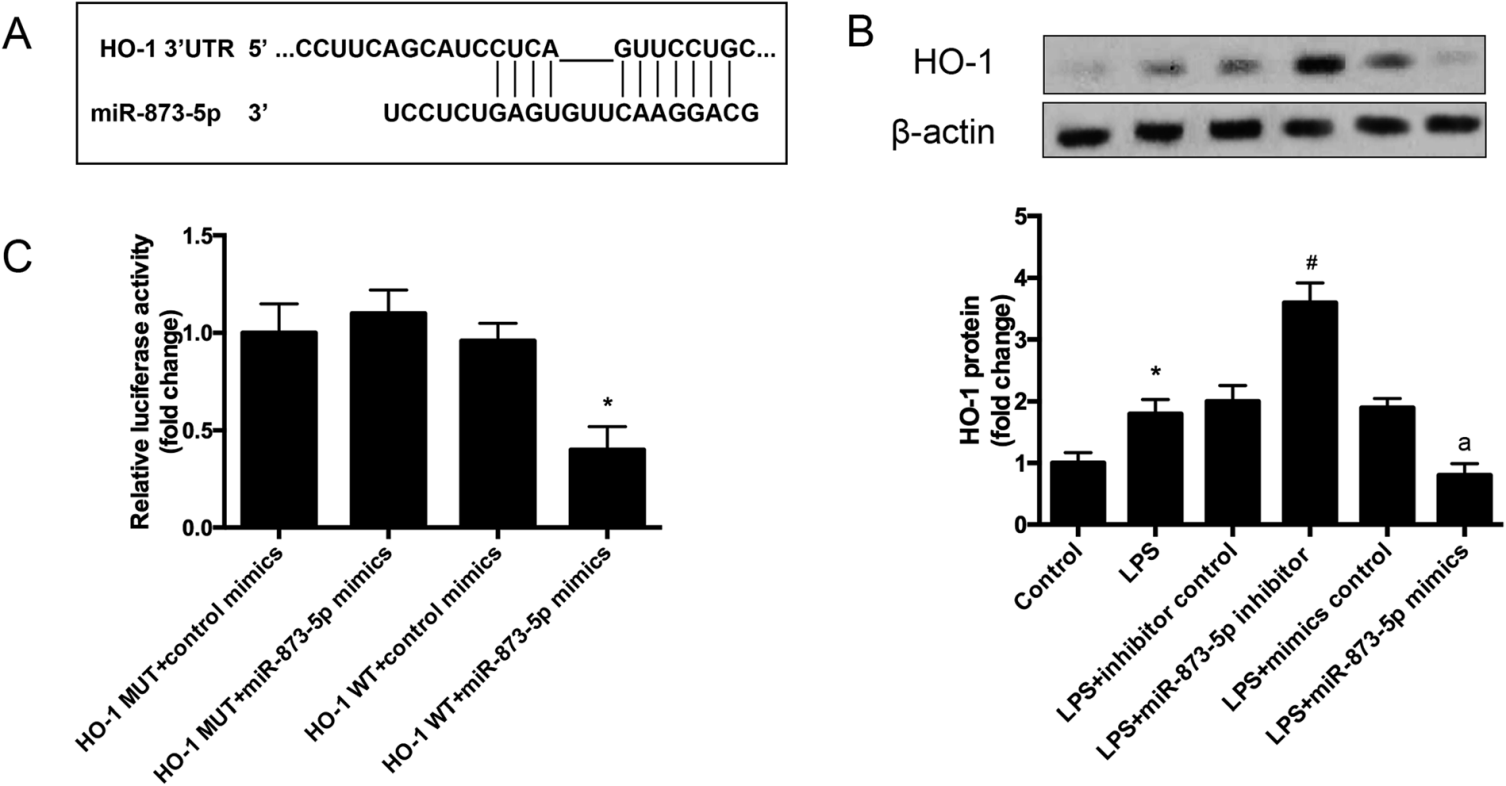
MiR-873-5p directly targeted to HO-1. (A) The target gene of miR-873-5p was predicted by an online software targetscan 7.2 (http://www.targetscan.org/vert_72/). (B) The expression level of HO-1 in KGN cells. The KGN cells was transfected with miR-873-5p inhibitor/inhibitor control or miR-873-5p mimics/mimics control, and then incubated in the presence or absence of LPS for 24 h. The HO-1 expression was analyzed by western blot. *p < 0.05 *vs.* control; ^#^*p* < 0.05 *vs.* LPS + inhibitor control; ^a^*p* < 0.05 *vs.* LPS + mimics control. (C) A luciferase reporter assay was performed to evaluate the interaction between miR-873-5p and HO-1. *p < 0.05 *vs.* HO-1 MUT + miR-873-5p mimics.

The target gene of miR-873-5p was predicted by an online software targetscan 7.2 (http://www.targetscan.org/vert_72/). Then the target genes were screened, and we found that one of the target genes, HO-1, is an important antioxidant enzyme. The predicted binding sites between miR-873-5p and HO-1 3′UTR were provided in Fig. 4A. We also found that LPS induced the expression level of HO-1 in KGN cells. The protein level of HO-1 was elevated in the cell transfected with miR-873-5p inhibitor, while HO-1 expression was inhibited by miR-873-5p mimics transfection, indicating that miR-873-5p might regulate the expression of HO-1 (Fig. 4B). Therefore, a luciferase reporter assay was performed to evaluate the interaction between miR-873-5p and HO-1. The results showed that the luciferase activity in cells co-transfected with miR-873-5p mimics and wide type 3′UTR fragment of HO-1 was significantly decreased compared with that in other groups (Fig. 4C).

### 3.4 HO-1 siRNA attenuated the effect of miR-873-5p inhibitor on oxidative stress in KGN cells

**Fig. 5:**
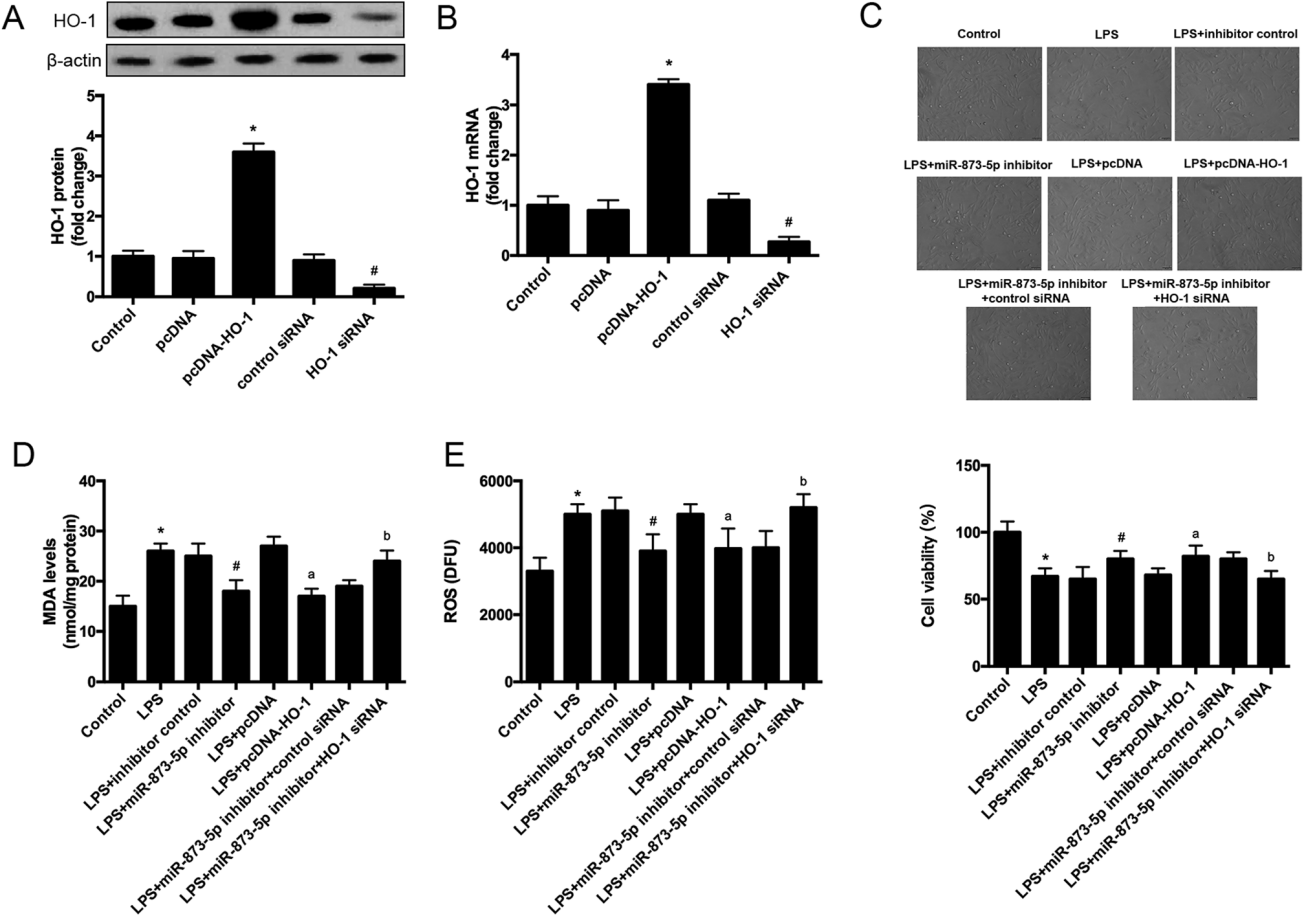
HO-1 siRNA attenuated the effect of miR-873-5p inhibitor on oxidative stress in KGN cells. To further investigate the role of HO-1 in the effect of miR-873-5p, the KGN cells were transfected with pcDNA–HO-1/pcDNA or HO-1 siRNA/control siRNA to change the HO-1 expression. (A and B) The protein and mRNA levels of HO-1 were measured by western blot and RT-PCR, respectively. *p < 0.05 *vs.* pcDNA; ^#^*p* < 0.05 *vs.* control siRNA. (C) Cell viability was measured by MTT assay. (D) MDA levels in KGN cells. (E) ROS production in KGN cells. *p < 0.05 *vs.* control; ^#^*p* < 0.05 *vs.* LPS + inhibitor control; ^a^*p* < 0.05 *vs.* LPS + pcDNA; ^b^*p*<0.05 *vs.* LPS + miR-873-5p inhibitor + control siRNA.

To further investigate the role of HO-1 in the effect of miR-873-5p in KGN cells, the cells were transfected with pcDNA–HO-1 or HO-1 siRNA to alter the HO-1 expression. As shown in Fig. 5A and B, the mRNA and protein levels of HO-1 were significantly increased in the cells transfected with pcDNA–HO-1, while were decreased in the cells transfected with HO-1 siRNA. HO-1 overexpression induced LPS-suppressed cell viability. Besides, HO-1 siRNA attenuated the induction effect of miR-873-5p inhibitor on cell viability in KGN cells (Fig. 5C). Moreover, HO-1 overexpression led to a decrease in the MDA and ROS production in LPS-induced KGN cells. HO-1 siRNA caused an increase in MDA and ROS production in the cells co-transfected with HO-1 siRNA and miR-873-5p inhibitor as compared to the cells transfected with miR-873-5p inhibitor only (Fig. 5D and E).

### 3.5 MAPK p38/Nrf2/HO-1 signaling pathway was implicated in the effect of miR-873-5p inhibitor in KGN cells

**Fig. 6:**
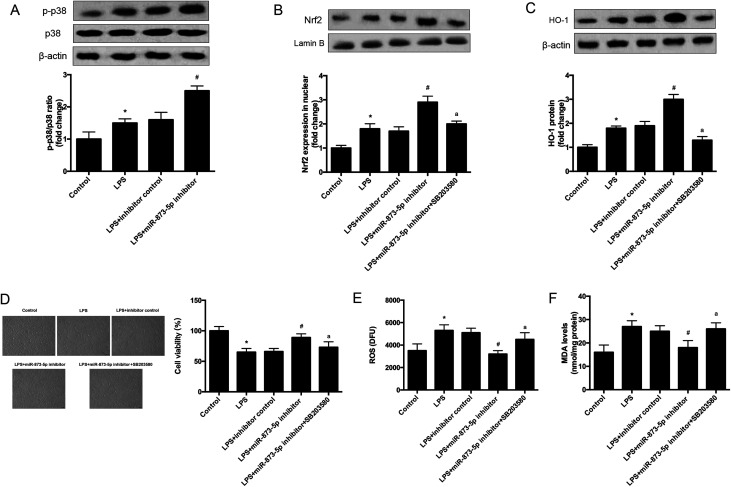
MAPK p38/Nrf2/HO-1 signaling pathway was implicated in the effect of miR-873-5p inhibitor in KGN cells. KGN cells was transfected with miR-873-5p inhibitor or inhibitor control, and then incubated in the presence or absence of LPS or SB203580 for 24 h. (A) The expression levels of p-p38 and p38 were detected by western blot. (B) The Nrf2 protein level in nuclear of KGN cells. (C) The expression levels of HO-1 in KGN cells. (D) Cell viability was measured by MTT assay. (E) ROS levels in KGN cells. (F) MDA production in KGN cells. *p < 0.05 *vs.* control; ^#^*p* < 0.05 *vs.* LPS + inhibitor control; ^a^*p* < 0.05 *vs.* LPS + miR-873-5p inhibitor.

It has been demonstrated that MAPK p38/Nrf2 signaling pathway plays an important role in response to oxidative stress.^15–17^ The expression levels of p-p38 and p38 were detected by western blot. As shown in Fig. 6A, the phosphorylation of p38 was induced by LPS stimulation in KGN cells, and the induction was enhanced by miR-873-5p inhibitor. Besides the effect of miR-873-5p inhibitor on Nrf2 was evaluated, and the western blot revealed that the Nrf2 protein level in nuclear was increased in LPS-induced KGN cells, and the miR-873-5p inhibitor enhanced the induction effect of LPS. However, the effect of miR-873-5p inhibitor on Nrf2 protein level in nuclear was inhibited by the inhibitor of p38 SB203580 (Fig. 6B). Moreover, HO-1 is a target gene of Nrf2, we also found that the increased HO-1 expression in miR-873-5p inhibitor transfected cell was suppressed by SB203580 (Fig. 6C). In addition, SB203580 also attenuated the effect of miR-873-5p inhibitor on cell viability, and production of ROS and MDA (Fig. 6D–F).

## 4. Discussion

PCOS is regarded as a chronic disease that closely associated with chronic inflammation and oxidative stress.^4^ Investigation of altered miRNAs expressions might reveal new targets for alleviating PCOS.^8^ MiR-873-5p has been reported to be an endocrine related miRNA that can be regulated by exogenous oxytocin in human myometrium.^18^ In the present study, we found that miR-873-5p levels were significantly up-regulated in follicular fluid from patients with PCOS. It has been revealed that the oxidative stress level is significantly increased in PCOS patients compared with normal individual.^4^ Oxidative stress is considered as a potential inducer for the pathogenesis of PCOS.^4^ Increasing evidences prove that oxidative stress in granulosa cells contributes to poor oocyte quality and results in PCOS.^19^ It is known that there is a close relationship between oxidative stress status and inflammation condition, elevated levels of oxidative stress usually results from and leads to an inflammatory response.^20^ It is difficult to separate inflammatory response from oxidative stress.^20^ Therefore, we used LPS to stimulate inflammatory response and oxidative stress in the KGN cells to simulate an *in vitro* model of PCOS as described previously.^14^ Interleukins such as IL-1β and IL-6 are identified as biomarkers of chronic inflammatory condition. ROS and MDA production and iNOS expression level are usually considered as markers of oxidative stress. We found that LPS induced the expression levels of IL-1β, IL-6 and iNOS, and induced the production of ROS and MDA, indicating LPS caused an inflammatory response and oxidative stress in KGN cells. And we found the miR-873-5p expression was up-regulated in the LPS-induced KGN cells. Besides, we also demonstrated that miR-873-5p inhibitor attenuated the LPS induced oxidative stress and cell apoptosis in KGN cells.

HO-1 is an important enzyme with potent anti-inflammatory and antioxidant properties that is induced in oxidative stress.^21^ It has been reported that the protein and mRNA levels of HO-1 in omental adipose tissue and human peripheral blood mononuclear cells (PBMCs) from the women with PCOS were significantly lower than those of the healthy controls.^22^ Gao *et al.* reported that women with PCOS present significantly increased levels of insulin resistance (IR), oxidative stress and inflammatory response.^23^ They also found that low level of HO-1 is associated with a higher risk for PCOS in non-obese women.^23^ In the current study, the results indicated that HO-1 is a target of miR-873-5p. The HO-1 expression was induced by LPS in KGN cells. However, HO-1 overexpression attenuated the oxidative stress induced by LPS in KGN cells. Besides, miR-873-5p inhibitor exhibited similar effect with HO-1 overexpression, and the HO-1 siRNA reversed the effect of miR-873-5p inhibitor on oxidative stress. The findings indicated that miR-873-5p regulated oxidative stress *via* targeting HO-1.

Nrf-2 is a transcription factor that regulates the expression of many antioxidant proteins, thus protecting cells from oxidative damage induced by injury and inflammation.^24,25^ The target genes of Nrf-2 include many cytoprotective proteins such as NAD(P)H quinone oxidoreductase 1 (Nqo1), UDP-glucuronosyltransferase (UGT) family, and glutathione *S*-transferase (GST) family.^24^ Besides, Nrf-2 has been known to induce expression of HO-1, and implicated with oxidative stress response.^24,26^ And the Nrf2/HO-1 pathway was observed to play a role in PCOS.^27^ It has been reported the MAPKs participates in the activation of Nrf2/HO-1 pathway.^28,29^ Lim *et al.* reported that p38MAPK/Nrf2 is activated to induce HO-1 expression as a response to ROS in vascular smooth muscle cell (VSMC).^30^ In the current study, we demonstrated that miR-873-5p inhibitor enhanced the activation of p38/Nrf2 signaling pathway induced by LPS. The inhibitor of p38, SB203580, also suppressed the HO-1 expression. We concluded that p38/Nrf2/HO-1 signaling pathway was implicated in the effect of miR-873-5p inhibitor on oxidative stress in KGN cells.

## 5. Conclusion

In summary, we evaluated the role of miR-873-5p and the potential molecular mechanism in PCOS *in vitro*. The results indicated that miR-873-5p was up-regulated in the follicular fluid from patients with PCOS and LPS-induced KGN cells. MiR-873-5p inhibitor attenuated the oxidative stress induced by LPS in KGN cells. Further investigations proved that miR-873-5p directly targeted HO-1. HO-1 siRNA alleviated the protective effect of miR-873-5p inhibitor on oxidative stress. Besides, we also found that p38/Nrf2/HO-1 signaling pathway was implicated in the effect of miR-873-5p inhibitor on oxidative stress in LPS-induced KGN cells. The findings provided a prominent insight into the roles of miR-873-5p in the pathogenesis of PCOS, indicating that miR-873-5p might be a therapeutic target for the prevention and treatment of PCOS complications.

## Conflicts of interest

The authors declare that there is no conflict of interest.

## Supplementary Material
