# Expression of concern: MiR-873-5p regulated LPS-induced oxidative stress *via* targeting heme oxygenase-1 (HO-1) in KGN cells

**DOI:** 10.1039/d2ra90009b

**Published:** 2022-02-09

**Authors:** Laura Fisher

**Affiliations:** Royal Society of Chemistry Thomas Graham House, Science Park, Milton Road Cambridge UK CB4 0WF advances-rsc@rsc.org

## Abstract

Expression of concern for ‘MiR-873-5p regulated LPS-induced oxidative stress *via* targeting heme oxygenase-1 (HO-1) in KGN cells’ by Hui Zhang *et al.*, *RSC Adv.*, 2018, **8**, 39098–39105. DOI: 10.1039/C8RA06697C

*RSC Advances* is publishing this expression of concern in order to alert readers that concerns have been raised over the integrity of the data published in this article. The authors have been contacted but have not responded to requests to provide raw data. An expression of concern will continue to be associated with the article until a conclusive outcome is reached.

Laura Fisher

31st January 2022

Executive Editor, *RSC Advances*

## Supplementary Material

